# A Retrospective Real-World Study: The Efficacy and Safety of Immune Checkpoint Inhibitors Combined with Chemoradiotherapy in Limited-Stage Small Cell Lung Cancer

**DOI:** 10.32604/or.2025.070893

**Published:** 2026-01-19

**Authors:** Ruoxue Cai, Shuyi Hu, Feiyang Li, Huanhuan Sha, Guoren Zhou, Ying Fang

**Affiliations:** 1 Department of Oncology, Geriatric Hospital of Nanjing Medical University, Jiangsu Province Official Hospital, The Affiliated Cancer Hospital of Nanjing Medical University, Nanjing, 210009, China; 2 Department of Oncology, The Affiliated Cancer Hospital of Nanjing Medical University, Jiangsu Cancer Hospital, Jiangsu Institute of Cancer Research, Nanjing, 210009, China; 3 Department of Oncology, Jiangsu Cancer Hospital, Jiangsu Institute of Cancer Research, The Affiliated Cancer Hospital of Nanjing Medical University, Nanjing, 210009, China

**Keywords:** Limited-stage small cell lung cancer, immunotherapy, chemoradiotherapy, TNM stage, type 2 diabetes

## Abstract

**Objective:**

To determine whether immunotherapy can bring new hope for patients with limited-stage small-cell lung cancer (LS-SCLC). We conducted this retrospective study to evaluate whether immunotherapy can achieve better efficacy in LS-SCLC patients.

**Methods:**

We evaluated 122 LS-SCLC patients who received concurrent chemoradiotherapy (CCRT) or sequential chemoradiotherapy (SCRT) (Group A) and immunotherapy combined with CCRT/SCRT followed by immunotherapy (Group B), to assess the objective response rate (ORR), disease control rate (DCR), and progression-free survival (PFS). Factors affecting prognosis were also explored using Cox analysis. The prognosis of patients with type 2 diabetes and patients with different TNM stages was compared to guide the selection of clinical regimens.

**Results:**

The overall ORR was 55.93%. The overall DCR was 98.31%. The DCR was 100% in Group A and 96.61% in Group B. There was no statistical difference in ORR and DCR. The overall median PFS was 9.86 months (95% CI, 8.62–11.10), and the difference in median PFS between the two groups was statistically significant (8.94 vs. 11.89 months, *p* = 0.03). The Cox regression analysis showed type 2 diabetes was associated with the survival prognosis. Patients with type 2 diabetes tended to choose immunotherapy combined with CCRT/SCRT. Patients in TNM stage IIIB had a significantly worse prognosis than those in stage I + II + IIIA.

**Conclusion:**

We suggest that LS-SCLC patients who receive immunotherapy combined with CCRT/SCRT can achieve longer PFS than those with CCRT/SCRT. Type 2 diabetes and TNM stage affect the survival prognosis. Patients with type 2 diabetes may benefit from immunotherapy combination treatments.

## 1 Introduction

Lung cancer is one of the major diseases affecting human survival in the world. Among them, small cell lung cancer (SCLC) accounts for 15%–17% of lung cancer. It has always been a perplexing clinical problem due to its high degree of malignancy, invasiveness, and susceptibility to recurrence and metastasis [1]. According to the Veterans Administration Lung Cancer Study Group (VALSG) staging, SCLC was divided into limited-stage and extensive-stage. Limited stage small cell lung cancer (LS-SCLC) means that the tumor is confined to metastasis in one side of the thorax, ipsilateral hilar, ipsilateral mediastinum, ipsilateral supraclavicular lymph nodes, and malignant pericardial effusion or malignant pleural effusion is excluded. Other than that, it is defined as extensive-stage small-cell lung cancer (ES-SCLC) [2,3].

With the advance of the immune era, more and more large clinical studies are demonstrating the effectiveness of immunotherapy in ES-SCLC and its superior efficacy compared to chemotherapy. The IMpower133 study was groundbreaking in finding that the Programmed Cell Death Ligand 1 (PD-L1) inhibitor Atezolizumab in combination with chemotherapy was significantly superior to chemotherapy for the first-line treatment of ES-SCLC patients [4], Median overall survival (OS) was 12.3 months in Atezolizumab + Etoposide + Carboplatin group compared with 10.3 months in the placebo + Etoposide + Carboplatin group (*p* = 0.007). Median progression-free survival (PFS) was 5.2 and 4.3 months, respectively (HR = 0.77; 95% CI 0.62–0.96; *p* = 0.02). The findings of this study have since raised the curtain on immunotherapy in SCLC. The subsequent CASPIAN study, ASTRUM-005 study, and CAPSTONE-1 study have successively confirmed the remarkable efficacy of immunotherapy in ES-SCLC [5–7].

However, the current clinical treatment for LS-SCLC patients is still limited to chemotherapy. Although the addition of radiotherapy has prolonged the survival of LS-SCLC patients to a certain extent, the survival time of these patients is still limited [8].

The STIMULI study found that patients with LS-SCLC who received consolidation therapy with Ipilimumab + Nivolumab after chemoradiotherapy (CRT) achieved a higher rate of adverse events than those who received CRT. Due to toxicities and treatment termination failed to observe effective treatment outcomes [9]. The ADRIATIC study provided a solid foundation for adjuvant therapy with Durvalumab for LS-SCLC patients following CRT. The study found that LS-SCLC patients who had no progressive disease (PD) after CRT received adjuvant therapy with Durvalumab had a median PFS of 16.6 months, which was significantly better than the placebo group (*p* = 0.02) [10]. As early as 2020, a single-arm clinical trial of Pembrolizumab and CRT in LS-SCLC patients found the triple therapy well-tolerated and with a longer PFS [11]. Subsequently, in 2022, a single-arm phase II study also found that Durvalumab with CRT for LS-SCLC exhibited promising clinical efficacy with a tolerable safety profile [12]. These studies above have paved the way for the clinical use of Immune checkpoint inhibitors (ICIs) in patients with LS-SCLC. Therefore, it deserves further exploration whether the clinical efficacy of triple therapy in LS-SCLC patients receiving ICIs combined with CRT, followed by ICIs adjuvant therapy, is superior to CRT.

Previous studies have shown that people with type 2 diabetes have a significantly higher risk of developing cancer than the general population [13]. Type 2 diabetes is strongly associated with the clinical efficacy of ICIs [14]. Patients with type 2 diabetes treated with ICIs have worse outcomes than patients without diabetes, which may be due to the effects of glucose-lowering medications. Patients with type 2 diabetes who are not receiving glucose-lowering medications have higher neutrophil-to-lymphocyte ratios [14]. Complex metabolic changes in patients with type 2 diabetes may produce multiple immunosuppressive effects that interfere with the efficacy of ICIs [15]. Previous studies have demonstrated that hyperglycemia induces neutrophil dysfunction, reduces the phagocytic activity of macrophages, and interferes with the antitumor effects of natural killer (NK) cells [16,17]. However, it has also been found that metformin can inhibit tumor cell proliferation by blocking part of the cell cycle [18] and exhibits synergistic antitumor effects when co-administered with the anti-PD-L1 inhibitor Atezolizumab [19]. Therefore, it is worthwhile to explore which treatments should be chosen for LS-SCLC patients with type 2 diabetes.

Significant associations have been reported between Body mass index (BMI) and survival risk for breast, rectal, pancreatic, and endometrial cancers. The risk of death in patients with these tumors increases with BMI [20,21]. BMI in tumor patients has also been associated with the efficacy of immunotherapy, with hypercholesterolemia and obesity decreasing the anti-tumor immune response of T-cells by affecting metabolism in tumor patients [19,22,23]. Thus, whether BMI in patients with LS-SCLC correlates with their survival prognosis deserves our attention.

For patients with LS-SCLC who achieve effective tumor control (complete response or partial response) following concurrent chemoradiotherapy, prophylactic cranial irradiation (PCI) significantly reduces the risk of brain metastases and prolongs survival. It is particularly recommended for stage II–III patients [24,25].

The VASLG method has been applied clinically to stage small-cell lung cancer [1]. However, it has been indicated that TNM staging can predict the likelihood of distant metastasis, brain metastasis, and survival in LS-SCLC. The risk of brain metastasis is lower in stage I and II SCLC than in patients with stage III SCLC. TNM staging guides as to whether prophylactic cranial irradiation should be applied to treat early-stage disease [26,27]. Whether TNM staging can further serve as a guide for treating LS-SCLC patients needs to be explored.

## 2 Methods

### 2.1 Study Population

This is a retrospective clinical study. We collected clinical data from electronic medical records of SCLC patients who were treated in Jiangsu Provincial Cancer Hospital from September 2021 to August 2024, and we screened the data related to LS-SCLC patients based on the medical records and VASLG staging. We collected general information, medical records, test results, imaging, pathology, and genetic testing data. We tracked outcomes based on existing clinical data up to the cutoff date for follow-up. Survival data of patients with LS-SCLC who received concurrent chemoradiotherapy (CCRT) or sequential chemoradiotherapy (SCRT) (Group A), ICIs combined with CCRT/SCRT followed by ICIs adjuvant therapy (Group B) were analyzed. Furthermore, no overlap in treatment regimens occurred between the two patient groups. Immune checkpoint inhibitors include PD-1 inhibitors and PD-L1 inhibitors. The chemotherapy regimen consists of etoposide plus platinum-based agents. Every patient who chose immunotherapy did so voluntarily. Our study adhered to the Declaration of Helsinki. This study was approved by the Ethics Committee of the Affiliated Cancer Hospital of Nanjing Medical University (No. 2022.259). Informed consent was obtained from each subject.

### 2.2 Research Endpoints

Each patient completed at least 2 cycles of one of these two regimens and was observed for 4 weeks or more. Imaging assessments of treatment efficacy are conducted after every 2–3 treatment cycles. We have documented that for some patients whose tumors are effectively controlled (CR or PR) following concurrent chemoradiotherapy, PCI is clinically administered. According to The Response Evaluation Criteria In Solid Tumors version 1.1 (RECIST v1.1), the short-term efficacy was assessed based on imaging results compared with the baseline. We evaluated recent clinical outcomes in real-world patients after receiving two therapies, with response to treatment categorized as complete response (CR), partial response (PR), stable disease (SD), and disease progression (PD). Progression-free survival (PFS) indicates the time from the start of receiving both treatment regimens to the disease progression occurs. If a patient stops treatment for a non-disease-progressive reason, such as an adverse reaction, the date of the last treatment is used as the cut-off time. Overall survival (OS) was not followed in this study because many patients in Group B had not yet experienced disease progression or died during the follow-up time. So the current OS results may be biased. We will continue to follow up. We were looking forward to subsequent updates on OS results.

### 2.3 Statistical Analysis

The clinical characteristics and adverse events were summarized by descriptive statistical analysis. Survival curves were plotted using the Kaplan-Meier. The efficacy between different groups was compared using the log-rank test (Log-Rank) method. Multiple variables affecting prognosis were analyzed by Cox regression analysis to explore factors affecting survival prognosis related to patients’ PFS. We also analyzed the PFS in the subgroup of patients with type 2 diabetes and different TNM stages. The study stipulated that *p* < 0.05 was considered statistically significant. The data obtained were analyzed using SPSS Statistics 26.0 software (Manufacturer Name: IBM Corporation, Corporate Headquarters Address: IBM Corporation, Armonk, NY, 10504, United States). We used GraphPad Prism 9.5 software (GraphPad Software, Inc., San Diego, CA, USA) for graphing.

## 3 Results

### 3.1 Patient Characteristics

We finally screened 122 patients with LS-SCLC from Jiangsu Cancer Hospital, who received one of the two therapies. Sixty-one patients were in Group A, and the other 61 patients were in Group B. None of these 122 patients had received surgical treatment after diagnosis. The detailed clinical characteristics of these 122 patients are shown in Table 1.

**Table 1:** Clinical characteristics of 122 patients

Characteristics	N(%)		
	All (N = 122)	Group A (N = 61)	Group B (N = 61)
**Age**			
<65	63 (51.64)	34 (55.74)	29 (47.54)
≥65	59 (48.36)	27 (44.26)	32 (52.46)
**Sex**			
Male	101 (82.79)	52 (85.25)	49 (80.33)
Female	21 (17.21)	9 (14.75)	12 (19.67)
**Smoking history**			
Yes	37 (30.33)	19 (31.15)	18 (29.51)
No	85 (69.67)	42 (68.85)	43 (70.49)
**ECOG score**			
0–1	107 (87.70)	54 (88.52)	53 (86.89)
2	15 (12.30)	7 (11.48)	8 (13.11)
**Type 2 diabetes**			
Yes	19 (15.57)	11 (18.03)	8 (13.11)
No	103 (84.43)	50 (81.97)	53 (86.89)
**Hypertension**			
Yes	40 (32.79)	19 (31.15)	21 (34.43)
No	82 (67.21)	42 (68.85)	40 (65.57)
**Initial BMI**			
≥25	50 (40.98)	24 (39.34)	26 (42.62)
<25	72 (59.02)	37 (60.66)	35 (57.38)
**Initial TNM**			
IB	3 (2.46)	1 (1.64)	2 (3.28)
IIA	5 (4.10)	3 (4.92)	2 (3.28)
IIB	32 (26.23)	16 (26.23)	16 (21.31)
IIIA	27 (22.13)	12 (19.67)	15 (22.95)
IIIB	55 (45.08)	29 (47.54)	26 (42.62)
**Radiotherapy site**			
Primary lung lesion + localized lymph nodes	122 (100.00)	61 (100.00)	61 (100.00)
Prophylactic cerebral irradiation	22 (18.03)	11 (18.03)	11 (18.03)

Note: ECOG, Eastern Cooperative Oncology Group; Group A, concurrent chemoradiotherapy (CCRT) or sequential chemoradiotherapy (SCRT); Group B, Immune checkpoint inhibitors combined with CCRT/SCRT, followed by Immune Checkpoint Inhibitors (ICIs) adjuvant therapy; BMI, Body Mass Index; ICI regimens: Anti-PD-1/PD-L1 antibodies; Relationship of radiotherapy with immunotherapy, concurrent chemoradiotherapy or sequential chemoradiotherapy; Radiotherapy dose: Primary lung lesion + localized lymph nodes: 45 Gy/30 f bid. Prophylactic cerebral irradiation: 25 Gy/10 f.

### 3.2 Assessment of Efficacy

#### 3.2.1 Short-Term Efficacy Assessment

Specific efficacy assessment based on RECIST v1.1 criteria of the imaging data of 118 patients whose imaging data could be traced throughout the whole process revealed that no patient reached CR, 66 patients reached PR, and 50 patients reached SD, with an overall ORR of 55.93% and an overall DCR of 98.31%. Among them, 33 patients reached PR and 26 patients reached SD in Group A. 33 patients reached PR, 24 patients reached SD, and 2 patients were PD in Group B. No statistically significant difference was observed between the ORR and DCR in these groups. The recent treatment responses of these 118 patients are detailed in Table 2.

**Table 2:** Response to treatment in all patients

Patients	Response—N (%)				ORR	DCR
	CR	PR	SD	PD		
Total patients (n = 118)	0 (0.00)	66 (55.93)	50 (42.37)	2 (1.70)	55.93%	98.31%
A (n = 59)	0 (0.00)	33 (55.93)	26 (44.07)	0 (0.00)	55.93%	98.31%
B (n = 59)	0 (0.00)	33 (55.93)	24 (40.68)	2 (3.39)	55.93%	96.61%
*p*					>0.999	0.154

Note: Objective response rate (ORR) = Complete response (CR) + Partial response (PR); Disease control rate (DCR) = Complete response (CR) + Partial response (PR) + Stable disease (SD); A, Group A: chemoradiotherapy (CCRT) or sequential chemoradiotherapy (SCRT); B, Group B: immunotherapy combined with CCRT/SCRT followed by immunotherapy; PD, Progressive Disease.

#### 3.2.2 Long-Term Efficacy Assessment

**Figure 1:**
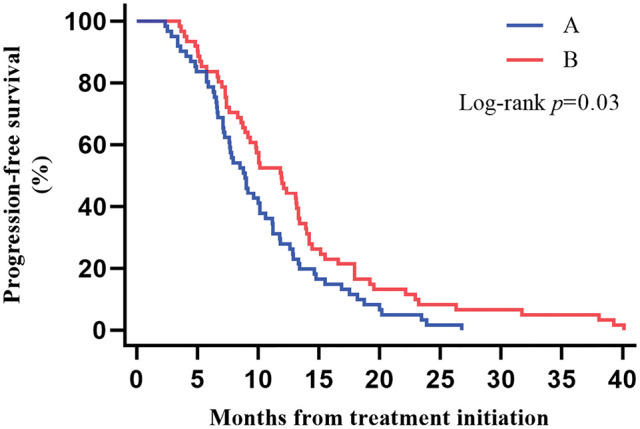
Kaplan–Meier estimates of progression-free survival (PFS) among the two groups

The median PFS was 9.86 months (95% CI, 8.62–11.10) for all patients, 8.94 months (95% CI, 7.43–10.45) for patients in Group A, and reached 11.89 months (95% CI, 9.54–14.24) for Group B patients. We found that patients with ICIs combined with CCRT/SCRT followed by ICIs adjuvant therapy can achieve longer PFS, and the difference was statistically significant (*p* = 0.03) (Fig. 1).

#### 3.2.3 Prognostic Factors for PFS

**Figure 2:**
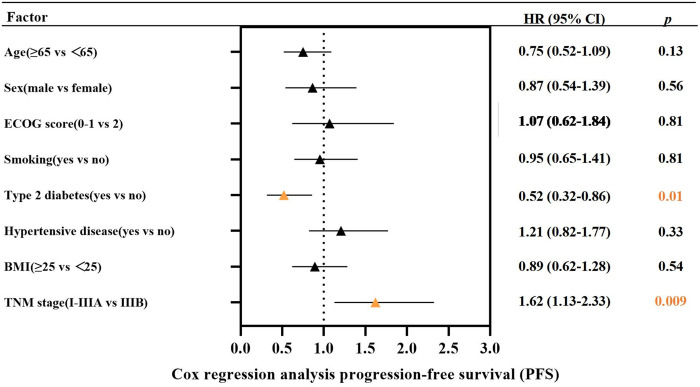
The Cox regression analysis for progression-free survival (PFS)

This study further explored the factors affecting PFS in these 122 patients by Cox regression analysis. As shown in Fig. 2, Cox regression analysis suggested that type 2 diabetes and TNM stage were associated with PFS in LS-SCLC patients. Such patients with type 2 diabetes had a poorer survival prognosis. No significant association between BMI and the survival prognosis of such patients has been found.

#### 3.2.4 Assessment of Long-Term Outcomes in Type 2 Diabetes Patients

**Figure 3:**
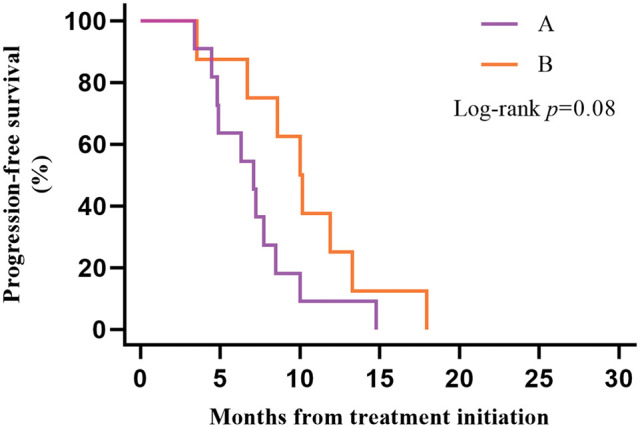
Kaplan–Meier estimates of progression-free survival (PFS) among patients in the type 2 diabetes group

A subgroup analysis of survival data from 19 LS-SCLC patients with type 2 diabetes found that 11 patients in Group A and 8 in Group B. Plotting KM survival curves on the PFS data of these patients revealed that, although there was no statistically significant difference (*p* = 0.08), patients receiving ICIs combined with CCRT/SCRT followed by ICIs adjuvant therapy (median PFS 10.02 months, 95% CI 7.83–12.21) tended to achieve a better outcome than patients receiving CCRT/SCRT (median PFS 7.10 months, 95% CI 4.59–9.61) with better clinical outcomes (Fig. 3).

#### 3.2.5 Evaluation of Efficacy in Patients with Different TNM Staging

**Figure 4:**
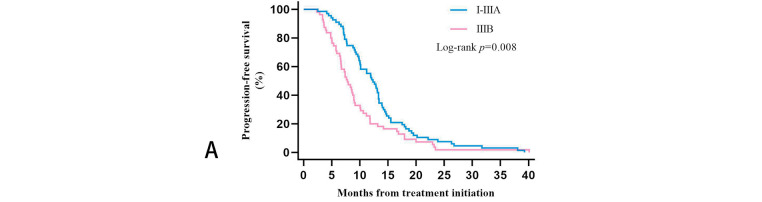

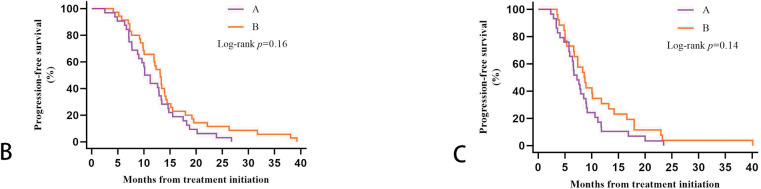
Kaplan-Meier estimates progression-free survival (PFS) among patients in different TNM stages: (**A**): PFS among stage I-IIIA and stage IIIB. (**B**): PFS in Groups A and B of stage I-IIIA patients. (**C**): PFS in Groups A and B of stage IIIB patients

We further clarified the specific TNM staging of 122 patients before receiving treatment by specifically evaluating the initial imaging data. Three patients were in stage IB, five in stage IIA, 32 in stage IIB, 27 in stage IIIA, and 55 in stage IIIB. After evaluating the PFS data of patients with these five stages separately, we found that the 55 patients with stage IIIB had the worst survival prognosis. Their PFS was significantly shorter than the 67 patients with the other four stages, and the difference was statistically significant (*p* = 0.008). After Bonferroni correction, *p* = 0.016, the difference between the two groups remained statistically significant. Twenty-nine of the 55 patients with stage IIIB in Group A, and 26 patients in Group B. Plotting the KM survival curves showed that although there was no statistical difference (*p* = 0.14), stage IIIB patients treated with Group B regimens tended to have longer PFS. However, both stage I-IIIA patients (*p* = 0.16) and stage IIIB patients were more likely to benefit from ICIs combined with CCRT/SCRT followed by ICIs adjuvant therapy (Fig. 4).

### 3.3 Adverse Events

We counted the common adverse reactions due to antitumor therapy during treatment in these 122 patients. The most common adverse reactions were anemia, leukopenia, and thrombocytopenia. 34 of the 122 patients experienced Grade 3–4 adverse reactions. Five patients discontinued the drug due to adverse reactions. More adverse events occurred in group B, the most common being anemia and thrombocytopenia, which were within manageable limits. No treatment-related deaths were observed. Existing clinical data indicate that immune-related adverse events include immune-mediated pneumonia, hypothyroidism, Hepatitis, Myocarditis, and hypopituitarism. Due to limitations in the available clinical data, adverse events recorded in our statistics may be incomplete. However, based on current information, the incidence of immune-related adverse events, such as immune-related pneumonia, is higher in Group B than in Group A (Table 3).

**Table 3:** Adverse events

Events	Grade 1–2		Grade 3–4	
	A (N = 61)	B (N = 61)	A (N = 61)	B (N = 61)
Overall adverse event	35 (57.38%)	40 (65.53%)	18 (29.51%)	16 (26.23%)
Leukopenia	17 (27.87%)	12 (19.67%)	5 (8.20%)	2 (3.27%)
Neutropenia	5 (8.20%)	0 (0.00%)	5 (8.20%)	7 (11.48%)
Thrombocytopenia	11 (18.03%)	13 (21.31%)	3 (4.92%)	3 (4.92%)
Anemia	32 (52.46%)	38 (62.30%)	4 (6.56%)	1 (1.64%)
Elevated aminotransferases	0 (0.00%)	0 (0.00%)	0 (0.00%)	1 (1.64%)
Fever	1 (1.64%)	2 (3.27%)	0 (0.00%)	0 (0.00%)
Rash	0 (0.00%)	0 (0.00%)	0 (0.00%)	0 (0.00%)
Vomiting	3 (4.92%)	1 (1.64%)	0 (0.0%)	0 (0.00%)
Anorexia	3 (4.92%)	1 (1.64%)	0 (0.00%)	1 (1.64%)
Numbness of the limbs	0 (0.00%)	0 (0.00%)	0 (0.00%)	0 (0.00%)
Proteinuria	1 (1.64%)	1 (1.64%)	1 (1.64%)	1 (1.64%)
Epistaxis	0 (0.00%)	0 (0.00%)	0 (0.00%)	0 (0.00%)
Thrombosis	0 (0.00%)	0 (0.00%)	0 (0.00%)	0 (0.00%)
Pneumonia	0 (0.00%)	3 (4.92%)	0 (0.00%)	1 (1.64%)
Hypopituitarism	0 (0.00%)	0 (0.00%)	0 (0.00%)	0 (0.00%)
Hypothyroidism	0 (0.00%)	0 (0.00%)	0 (0.00%)	0 (0.00%)
Hepatitis	0 (0.00%)	0 (0.00%)	0 (0.00%)	1 (1.64%)
Myocarditis	0 (0.00%)	0 (0.00%)	0 (0.00%)	0 (0.00%)

Note: Grade 1–2, Grade 1–2 adverse events; Grade 3–4, Grade 3–4 adverse events.

## 4 Discussion

This study found that ICIs combined with CCRT/SCRT followed by ICIs adjuvant therapy had a better clinical outcome than CCRT/SCRT in LS-SCLC patients, i.e., significantly longer PFS. Type 2 diabetes is strongly associated with the survival prognosis of LS-SCLC patients. In particular, LS-SCLC patients with type 2 diabetes who received ICIs combined with CCRT/SCRT followed by ICIs adjuvant therapy tended to have a better clinical outcome than those who received CCRT/SCRT. No significant survival difference existed between patients with BMI ≥25 kg/m^2^ and those with BMI <25 kg/m^2^. The difference in initial BMI did not lead to a different survival prognosis. The LS-SCLC patients in the IIIB stage had a worse survival prognosis than patients with other stages.

The clinical treatment for LS-SCLC has been chemotherapy with etoposide combined with platinum drugs. At the same time, cwhen ombined with radiotherapy for lung lesions and regional lymph nodes, its efficacy is not satisfactory, with a 5-year survival rate of only 29% to 34% [8]. Prophylactic brain irradiation therapy increases clinical prognosis in LS-SCLC patients. However, the ultimate survival benefit remains limited [2].

Immune checkpoint inhibitors can reactivate T-cell anti-tumor immune responses by blocking immune-suppressive signaling pathways, such as PD-1/PD-L1. Meanwhile, radiotherapy can enhance tumor antigen presentation through immunogenic cell death, and chemotherapy drugs, such as etoposide, also possess certain immunomodulatory effects [28]. Various studies, including IMpower-133, CASPIAN, ASTRUM-005, and RATIONALE-312, have demonstrated that combining ICIs and chemotherapy significantly improves the prognosis of patients with ES-SCLC [5,6,29]. This ushered the treatment of SCLC into the era of immunotherapy. In recent years, clinical studies on the use of immunotherapy in LS-SCLC have been in full swing. In 2020, a single-arm clinical trial of Pembrolizumab and CRT in LS-SCLC patients also found the triple therapy to be well tolerated, with a median PFS of 19.7 months (95% CI: 8.8–30.5) and a median OS of 39.5 months (95% CI: 8.0–71.0) [11]. This study demonstrated that concurrent immunotherapy combined with chemoradiotherapy is safe and effective. In 2022, a phase II clinical trial found that the combination of Durvalumab with CRT in LS-SCLC showed good clinical efficacy (median PFS of 14.4 months, 24-month PFS rate of 42.0%) and tolerable safety. The efficacy advantage was not related to the expression level of PD-L1 [12]. After that, the ADRIATIC study conducted by Prof. Ying Cheng’s team at Jilin Cancer Hospital found that Durvalumab, as a consolidation therapy after CRT in non-progressed LS-SCLC patients, showed better clinical efficacy than placebo. It significantly prolongs the PFS and OS. This study breaks the 30-year-long deadlock in the treatment of LS-SCLC [10]. This prospective study investigates the clinical efficacy of ICIs consolidation therapy in LS-SCLC patients after CRT. However, the study did not compare the efficacy differences between immunotherapy combined with chemoradiotherapy and chemoradiotherapy alone. ASTRUM-020, a large-scale prospective study on the mode of immunotherapy advancement to CRT followed by immune adjuvant therapy, is still in the exploratory stage. A retrospective study published this year by Xinqing Lin’s team shows encouraging clinical efficacy and an acceptable safety profile for LS-SCLC patients treated with immunotherapy combined with CRT or chemotherapy alone in the first line [30]. Patients who received chemotherapy combined with immunotherapy had longer OS than patients treated with chemotherapy alone. However, this retrospective study has not yet compared the survival differences between immunotherapy and CRT vs. CRT alone.

Due to the synergistic anti-tumor effects of radiotherapy, immunotherapy, and chemotherapy [28,31–33], as well as positive results from some of the clinical trials mentioned previously, many LS-SCLC patients received ICIs combined with CRT, followed by ICIs adjuvant therapy. This retrospective study collected information about these patients and performed statistical analysis. We found that the survival prognosis of LS-SCLC patients treated with ICIs combined with CCRT/SCRT, followed by ICIs adjuvant therapy, was statistically better than CCRT/SCRT. The difference was statistically significant, and it was well tolerated. This study further validates the positive efficacy of immunotherapy combination regimens in localized SCLC. It pioneers the discovery that immunotherapy combined with chemoradiotherapy represents a superior treatment option compared to chemoradiotherapy alone. This will provide effective guidance for selecting treatment options in the future.

Previous studies have shown that metabolic changes such as hyperinsulinemia and insulin-like growth factor I, hyperglycemia, dyslipidemia, adipokines and cytokines, and intestinal microbes in patients with obesity and type 2 diabetes may contribute directly or indirectly to cancer progression. Thus, patients with obesity and type 2 diabetes are at increased risk of cancer and cancer-related death [34]. Numerous findings suggest that complex metabolic changes in patients with type 2 diabetes may produce multiple immunosuppressive effects that interfere with the efficacy of ICIs [14,16,17]. However, it has also been found that metformin, a glucose-lowering drug commonly used in patients with type 2 diabetes, can have a synergistic anti-tumor effect when combined with the PD-L1 inhibitor Atezolizumab [19]. In this study, we found that having type 2 diabetes was a factor affecting the survival prognosis of LS-SCLC patients, as determined by Cox regression analysis. Our further analysis in the subgroup of patients with type 2 diabetes found that, although there was no statistically significant difference (*p* = 0.08), such LS-SCLC patients with type 2 diabetes tended to survive with ICIs combined with CCRT/SCRT, followed by ICIs adjuvant therapy (median PFS 10.02 months, 95% CI 7.83–12.21) longer than such type 2 diabetic patients with CCRT/SCRT (median PFS 7.10 months, 95% CI 4.59–9.61) to achieve better clinical outcomes. It is believed that expanding the sample size of the study could have yielded even more statistically significant differences. We not only confirmed that type 2 diabetes was associated with a worse survival prognosis in LS-SCLC but also found that LS-SCLC patients with type 2 diabetes were more inclined to receive immunocombination therapy. Future prospective studies should account for the potential bias arising from the impact of diabetes as a variable on outcomes. Diabetic patients inherently exhibit immune dysfunction and microcirculatory disorders, making them a high-risk group for infections. This retrospective study has not identified a higher incidence of immune-related adverse events in diabetic patients receiving immunotherapy compared to those not receiving immunotherapy. In contrast, no significant difference in survival prognosis has been found between obese patients (BMI ≥ 25 kg/m^2^) and normal weight patients.

Tumor staging is a key factor in the prognosis and management of lung cancer patients. The VASLG has long been applied to SCLC. However, when the International Lung Cancer Association released the seventh edition of TNM staging, it began to recommend that TNM staging be used when staging SCLC patients [35]. The International Association for Lung Cancer (IALC), in its seventh edition of TNM staging, recommended the use of TNM staging in staging SCLC patients, and the design of clinical trials for early-stage disease stratified by stages I, II, and III [36]. The International Association for the Study of Lung Cancer (IASLC), after analyzing the survival of SCLC patients with clinical and pathological staging between 1999 and 2010, and analyzing the results of the latest eighth edition of TNM staging, found that the survival rate of patients with clinical stage T1 was significantly higher and was not related to surgery. There was almost no difference in the survival rate between T1a and T1b, and between T2a and T2b. The survival rate of T1 was better than that of T2, and T3 was better than T4. However, there was no significant survival difference between T2 and T3. Moreover, the survival rate of SCLC patients tended to decrease with the increase of clinical stage [3]. This suggests that the TNM stage may be associated with the clinical prognosis of SCLC patients. Due to the limited sample size, this study did not find survival differences between the various TNM stages. However, we found that patients with LS-SCLC in stage IIIB had a statistically significantly worse prognosis compared with patients in other stages. However, patients in stages I-IIIA and IIIB still preferred to receive ICIs combined with CCRT/SCRT, followed by ICIs adjuvant therapy to achieve a better clinical prognosis. The TNM stage is associated with clinical prognosis in LS-SCLC patients, but it cannot be used to guide the choice of the two treatment options.

This study has certain limitations. First, as a retrospective clinical study, the inadequate sample size may introduce data bias. Second, the study lacks long-term follow-up data on overall survival (OS), which we will continue to monitor. Third, the heterogeneity among different immune checkpoint inhibitors may lead to biased study results.

## 5 Conclusion

In conclusion, the results of this retrospective study suggest that LS-SCLC patients can achieve better clinical outcomes by receiving ICIs combined with CCRT/SCRT followed by ICIs adjuvant therapy. Type 2 diabetes and TNM stage affect survival prognosis in LS-SCLC patients, but differences in BMI were not associated with survival prognosis in LS-SCLC patients. Patients with type 2 diabetes may benefit from immunotherapy combination treatments, but this requires a larger sample size to support. TNM stage can not yet guide the treatment choice for LS-SCLC.

## Data Availability

Data are available upon reasonable request. Anonymized individual participant data and study documents can be requested for further research.
